# Validity of flounce sign to rule out medial meniscus tear in knee arthroscopy

**DOI:** 10.1186/s12891-015-0800-2

**Published:** 2015-11-06

**Authors:** Yogendra Gupta, Deepak Prakash Mahara, Arjun Prasad Lamichhane

**Affiliations:** Department of Orthopedics and Trauma, Nobel Medical College and Teaching Hospital, Biratnagar, Nepal; Department of Orthopedics and Trauma, Tribhuwan University-Teaching Hospital, Kathmandu, Nepal

**Keywords:** Arthroscopy, Flounce sign, Meniscus tear

## Abstract

**Background:**

The meniscal flounce is wavy fold in free inner border of meniscus seen during knee arthroscopy. The presence of this flounce in medial meniscus has been found to be highly predictive of normal medial meniscus. With meniscus related symptoms being commonest indication for undergoing knee arthroscopy, presence or absence of medial meniscus flounce, can be a good guiding sign. In this study, we aimed to validate the significance of the flounce sign in ruling out medial meniscus tear.

**Methods:**

A prospective study was undertaken to validate the significance of flounce sign. There were 62 patients who underwent arthroscopic surgery of the knee over the duration of one and half years. Free inner margin of medial meniscus as seen through anterolateral portal was recorded for the presence or absence of flounce. The sensitivity, specificity, positive predictive value (PPV), negative predictive value (NPV) and accuracy of this sign was then calculated for diagnosing normal medial meniscus. Significance was tested with chi square test with 95 % confidence interval.

**Results:**

A total of 62 cases were included. The sensitivity, specificity, PPV, NPV and accuracy of flounce sign was found to be 67.64 %, 92.85 %, 92 %, 70.27 % and 79.03 % respectively, and the result was significant (*p* value = 0.0001).

**Conclusion:**

The flounce sign has been shown to have high diagnostic value. Use of it in routine knee arthroscopy can be helpful, particularly during screening procedure and in exploring tears which are usually not seen easily through routine portals.

## Background

Lesions of the meniscus are frequently seen and arthroscopic meniscus surgery is the most commonly performed arthroscopic procedure in the knee joint [1]. The meniscal flounce is a fold in the free non-anchored inner margin of the meniscus [2]. Its presence is thought to be predictive of an intact meniscus [2–4].

The presence or absence of a flounce has been shown to have high sensitivity, specificity and predictive value for normal or torn medial meniscus by Wright RW et al. [3] and they suggest that the absence of flouncing on medial meniscus during arthroscopy could be valuable sign in guiding the operating surgeon to search for occult medial meniscus tear. Further, they have suggested that the importance of this sign is greatly increased in situations in which either the surgeon’s attention is concentrated on other associated injuries: the patient has a meniscus tear that is not readily identified: or the knee arthroscopy is being done as a part of screening procedure in conjunction with some other major procedure [3].

In this study, our aim was to validate the meniscus flounce sign for detecting presence or absence of medial meniscus tear during arthroscopy.

## Methods

This was a hospital based prospective observational study done in the patients undergoing arthroscopic surgery of the knee. There were 62 patients who underwent arthroscopic surgery for various reasons in the period of one and half year. Cases with previous partial meniscectomy, excessive fraying of the inner free border enough to make it difficult to visualize, discoid medial meniscus and medial meniscal tears in the presence of any grade of laxity of the medial collateral ligament were excluded from the study.

The study was started after approval from institutional review board (Maharajgunj Medical Campus, Institute of medicine, Tribhuwan University). Informed written consent was taken from all eligible patients before enrolling in the study. No extra financial burden was placed upon patient.

Patient enrolled in the study underwent short interview regarding their symptoms, mode of injury; if any, duration and other demographic questionnaires. The knee arthroscopies were undertaken by two fellowship trained surgeons (DPM, APL). None of the surgeons were blinded tothe clinical diagnosis or MRI findings, when available.

**Fig. 1:**
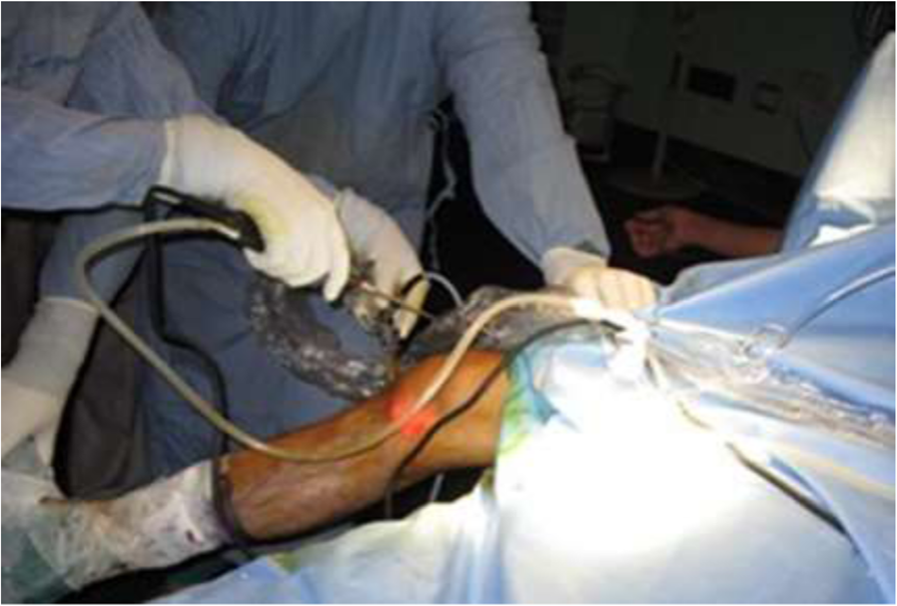
The position for viewing the medial meniscus in right knee

**Fig. 2:**
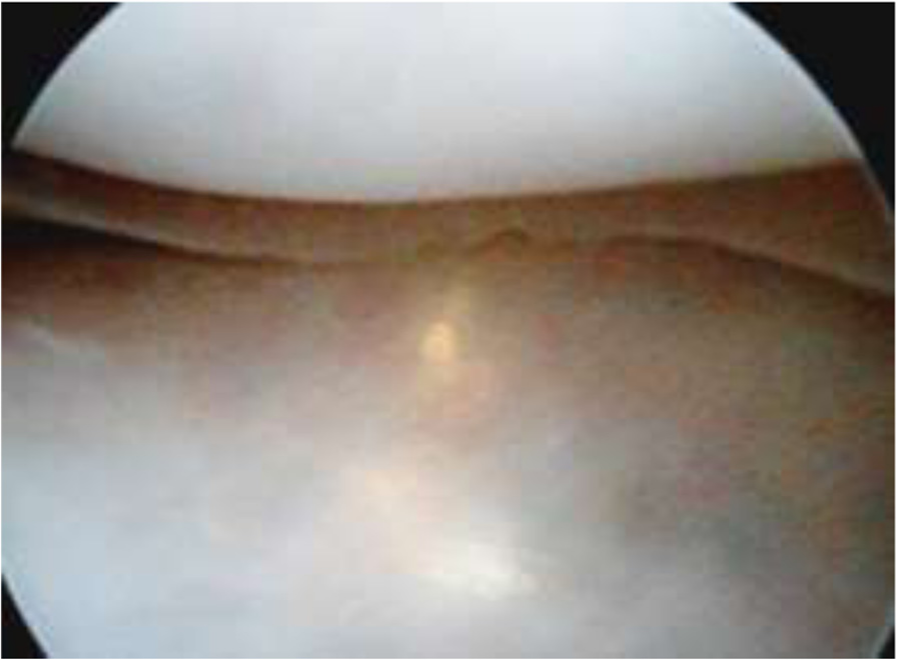
Appearance of medial meniscus flounce as seen through anterolateral portal, case illustration - 1

**Fig. 3:**
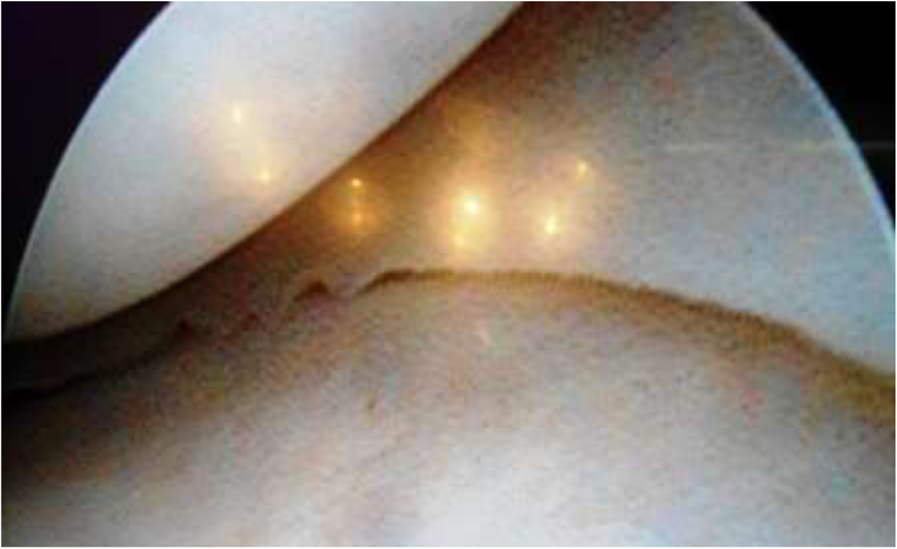
Appearance of medial meniscus flounce, case illustration - 2

**Fig. 4:**
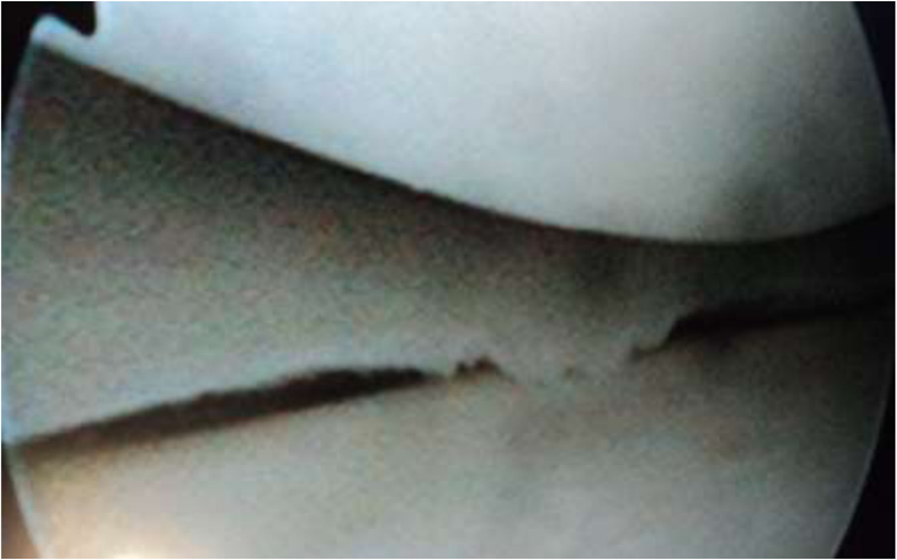
Appearance of medial meniscus flounce, case illustration - 3

**Fig. 5:**
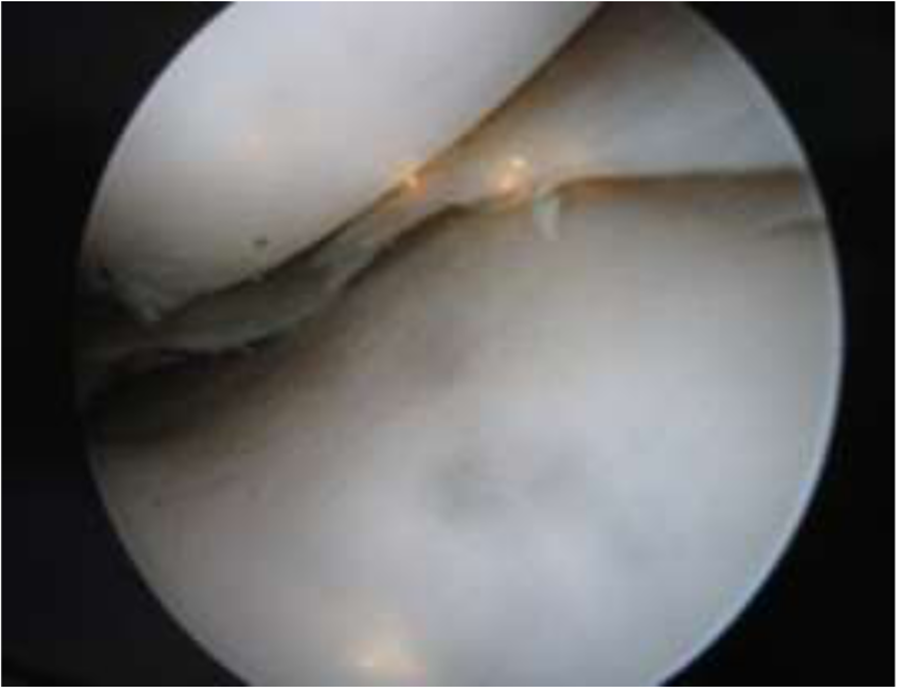
Medial meniscus without flounce as seen through anterolateral portal

**Fig. 6:**
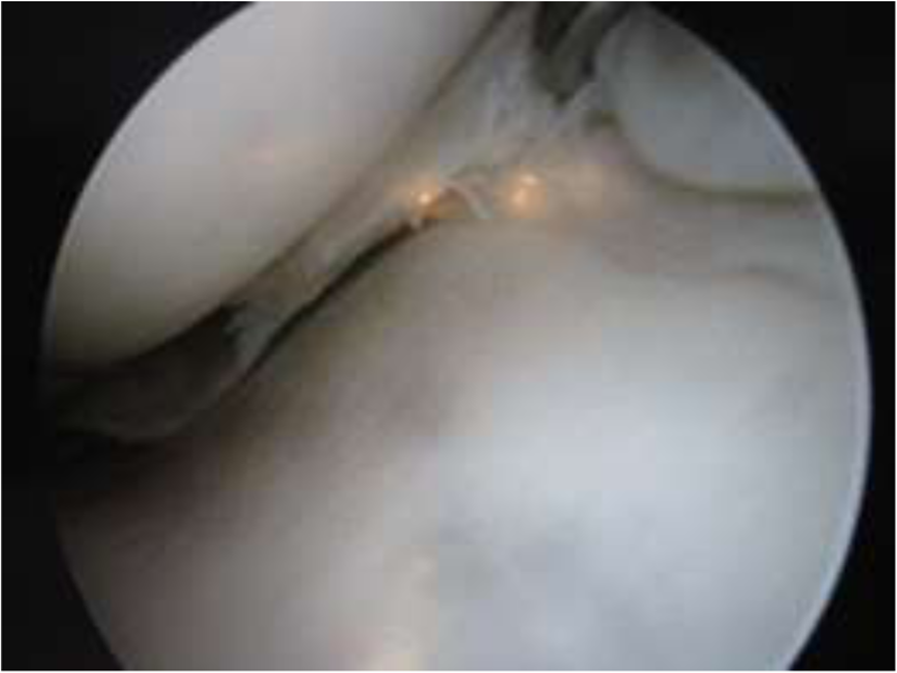
Tear in medial meniscus seen on probing in a case without flounce

Arthroscopy of the knee was performed with the patient supine, under general or spinal anesthesia, with a pneumatic tourniquet and under all aseptic precautions. Standard anterolateral and anteromedial portals were made in all cases. Scope was inserted via anterolateral portal and then knee was positioned in approximately twenty degree of flexion and a valgus and external rotation force was applied by accompanying assistant (Fig. 1). The appearance of free edge of medial meniscus was noted. If the view was obscured by fraying then shaving was done to make the vision clear. The presence (Figs. 2, 3 and 4) or absence of meniscal flounce sign (Fig. 5) was noted at this moment and recorded. Next, thorough visualization of all parts of medial meniscus as well as probing was done in all cases to confirm the absence or presence of medial meniscus tear and it was recorded (Fig. 6). All other compartments were visualized according to the standard guidelines and arthroscopy was carried out .

A result was considered to be true-positive (TP***)*** when the presence of meniscal flounce was accompanied by normal medial meniscus on arthroscopy. A false-positive (FP) result was defined as a presence of medial meniscus flounce along with medial meniscus tear on arthroscopy.

A result was considered true-negative (TN) when the absence of flouncing wasaccompanied with medial meniscus tear. A false-negative (FN) result was defined as absent flounce but normal medial meniscus.

The sensitivity, specificity, and positive predictive value (PPV), negative predictive value (NPV) and accuracy were then calculated by constructing two by two cross table. Analysis of data was done using the SPSS 16 package. The statistical significance of observations was tested by the Chi squared test and when needed logistic regression analysis was performed. *p* value ≤ 0.05 was considered significant.

## Results

Out of 62 patients 39 (63 %) were male and 23 (37 %) were female. Age of the patients ranged from 11 to 70 years with mean age of 27.35 years. Majority of the patients 46 (74.2 %) were of less than 30 years and only 3 patients 3 (4.8 %) were of more than 50 years of age. Injury pattern of our patients was sports in 21 (34 %) and non-sports 41 (66 %). Sports injury was found to be common mechanism in young patients. Patient presented to us with minimum duration of symptoms of 1 week and maximum of 96 months. Average duration of symptoms prior to presentation was 19.18 months. Twenty eight patients (45.20 %) underwent arthroscopy in left knee whereas 34 (54.80 %) underwent in right knee. The most common cause of presentation of our patients was pain, in 41 (36.60 %) patients. Other causes of presentation in decreasing order were giving away in 31 (27.70 %), swelling and locking each in 16 (14.30 %) and snapping symptoms in 6 (5.40 %) cases.

The sensitivity, specificity, PPV, NPV and accuracy of medial meniscal flounce sign in indicating normal or torn medial meniscus was calculated by forming 2 × 2 cross table (Table 1) and was found to be 68 %, 93 %, 92 %, 70 % and 79 % respectively, which was significant, as tested by chi square test (*p* value = 0.0001).

**Table 1:** Flounce sign and medial meniscus tear (arthroscopic diagnosis)

Medial meniscus flounce sign	Arthroscopic medial meniscus		Total
	Normal	Torn	
Positive	(TP) 23	(FP) 2	25
Negative	(FN) 11	(TN) 26	37
Total	34	28	62

Sensitivity of Test = TP/TP + FN = 23/34 = 0.6764

Specificity of Test = TN/TN + FP 26/28 = 0.9285

Positive Predictive Value = TP/TP + FP23/25 = 0.9200

Negative predictive value = TN/TN + FN = 26/37 = 0.7027

Accuracy = TP + TN/ALL CASES = 23 + 26/62 = 0.7903

The association of medial meniscus flounce sign and medial meniscus tear was further tested if any third variable like ACL or Lateral meniscus has any effect by applying logistic regression analysis. When tested with status of ACL (torn or intact, number of ACL torn 32) *p* value came to be 0.278 which was not significant. Similarly when tested with status of lateral meniscus (lateral meniscus torn cases 19) *p* value came to be 0.590 which is also not significant.

## Discussion

The meniscal flounce is a fold in the free, non-anchored inner edge of the medial meniscus [2–4]. It is like few small ripples in the free edge that disappear at one end. The appearance of the flounce has been reported at arthroscopy [2–4], arthrography [5] and MRI scanning [6–8].

The meniscal flounce is produced when stress maneuvers are applied to the tibiofemoral joint, resulting in distraction and some rotation of the compartment being assessed. During these stress maneuvers, meniscal motion and configuration are influenced by the capsular and ligamentous attachments, relative tibiofemoral movement and loading of the knee joint. When the meniscus is stressed, the peripheral attachments cause differential stresses within the body of the intact meniscus. These stresses manifest as buckling of the free inner margin of the meniscus known as the meniscal flounce. Pathology affecting the integrity of the meniscus or its attachments can alter the appearance of this flounce [2].

The meniscal flounce in knee arthroscopy was first described in an illustration in the Atlas of Arthroscopy by Watanabe et al. [4], the first such collection on the subject of arthroscopic anatomy. Though described very early in the literature, its significance was not studied till 2006, when Williams AM et al. [2] and in 2007, Wright RW et al. [3] showed that the presence of this very simple manifestation of stress maneuver and axial loading can be of great help owing to its high sensitivity and specificity in diagnosing normal meniscus. Williams AM et al. [2] not only established high diagnostic value but also tried to characterize the size location and characteristics of normal flouncing on medial meniscus and lateral meniscus.

Similar results as ours was seen in study by Williams AM et al. [2] in which they showed: for an intact medial meniscus the sensitivity, specificity, and positive predictive value (PPV) were 68.50 %, 92.9 %, and 92.10 % respectively. Conversely the presence of meniscal pathology correlated closely with either an absent or abnormal flounce (*P* <0.0001). In study by Wright RW et al. [3], the presence of a meniscal flounce sign had a positive predictive value of a normal medial meniscus of 0.97 (63/65 knees). Absence of the meniscal flounce sign had a positive predictive value of an abnormal meniscus of 0.98 (101/103 knees). Specificity was 98 % (101/103 knees), and sensitivity was 97 % (63/65 knees).

Our study had large number of false negative cases (17.72 %), meaning neither there were flounce on first view nor was tear on probing, giving low sensitivity and NPV. But our study had high specificity and PPV due to very low false positive cases. Since sensitivity denotes an ability of the test to rule out negative result, number of false negative cases decreases the diagnostic values. The results are comparable to the study by Williams AM et al. [2].

For the sign to have high sensitivity and NPV, false negative cases must be less. Though the surgeons doing arthroscopy in this study are trained Sports Fellow and experienced, interobserver fallacies can’t be ruled out. Difficulty in visualization especially of posterior third of medial meniscus is well established [9]. At the same time large number of tears are also found in the same zone. So it is very likely for tear in this zone to be missed despite thorough visualization. One way to minimize this inherent difficulty is to use accessory portals in cases of high suspicion (either clinically or if other diagnostic images like MRI suggests). We didn’t use any accessory portal for exploring any possible subtle medial meniscus tear when there was seemingly normal meniscus in absence of flouncing. Also in this study valgus and external rotation was given manually by an assistant so, there can be slight variation in the pattern of differential stress created in medial meniscus and so flouncing was not appreciated.

The influence of other associated intra-articular derangement like ACL insufficiency or associated lateral meniscus tear were tested by applying logistic regression analysis and was found to be insignificant (*p* value =0.278 for ACL and *p* value = 0.590 for Lateral meniscus status). Wright RW et al. [3] have also stated that there was no association between chondral injuries or other ligament injuries, while Williams AM et al. [2] have shown significant association between medial collateral ligament insufficiency and medial flouncing.

**Fig. 7:**
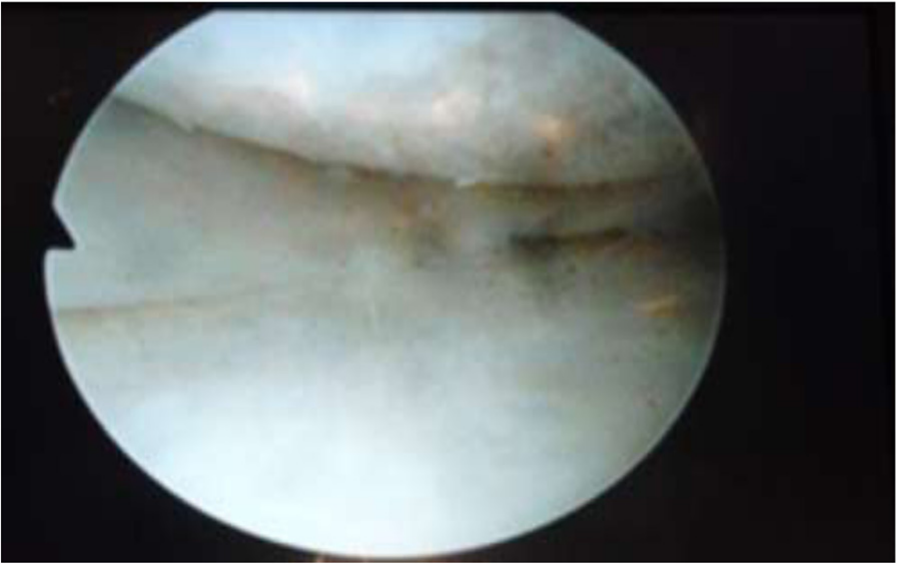
Medial meniscus free edge as seen in false positive case

**Fig. 8:**
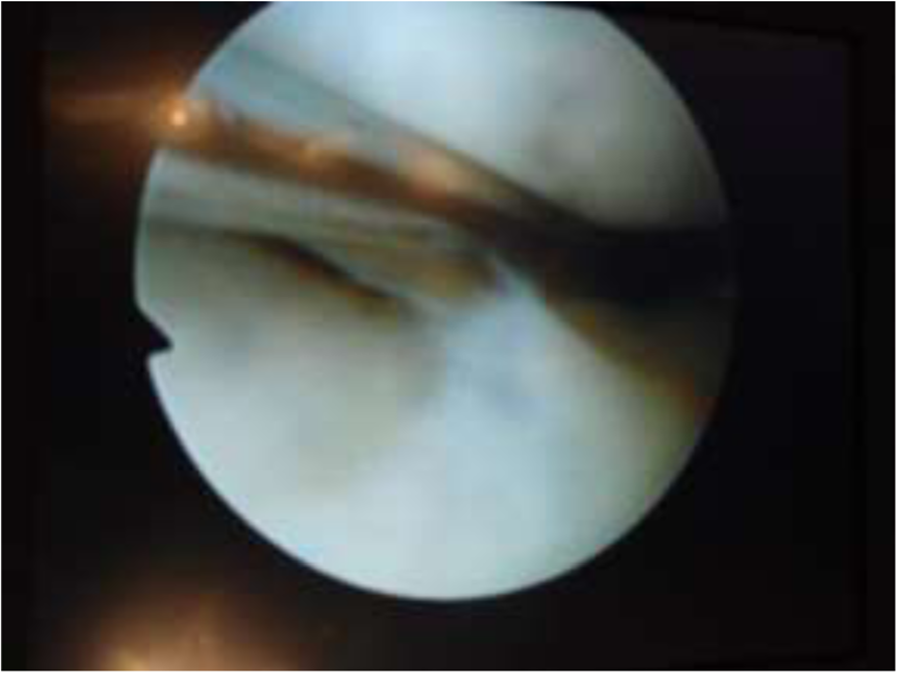
Tear seen on probing of false positive case

Low false positive cases (3.2 %) in our study increased the specificity and PPV, giving high diagnostic value to the test in diagnosing normal meniscus. One false positive case actually had a large wavy pattern without normally decreasing height of wave (Fig. 7). Probing of that case revealed tear (Fig. 8). Since we have not characterized the appearance or size in our study we included this case as false positive.

Since this is only one of the few studies of its kind, to best of our knowledge, usefulness of its high specificity and PPV can’t be ignored. Also, despite few false negative cases, accuracy of the sign is nearly 80 % which is fair enough for clinical purpose but needs validation in multiple studies, in multiple settings and with larger number of cases.

Potential observer bias regarding detecting meniscal injury in the medial compartment based on preoperative clinical and MRI findings, small sample size, study duration and variation in the ligament laxity in population are some of the limiting factors.

This study has established high specificity and PPV of the flounce sign with fair sensitivity, NPV and accuracy of the sign. This can be especially useful in two clinical settings. First, in cases which are clinically diagnosed with medial meniscus tear and are going for arthroscopic management, non visualization of tear with apparently normal looking medial meniscus and in the absence of other intra-articular derangement and absence of flouncing on inner edge, the surgeon can become more vigilant in searching for tear especially in areas like posterior third. If highly suspected clinically and diagnostic images like MRI support the diagnosis, the surgeon may use accessory portal to look for the tears. Secondly, this sign can be used to rule out medial meniscal tear while doing other knee procedures like ACL and PCL reconstruction and during surgery for patellofemoral instability. During such procedures, the presence of flouncing on inner edge of medial meniscus can give idea that the meniscus is normal and there by aggressive instrumentation can be avoided; limiting iatrogenic chondral injuries and reducing the surgical time significantly. At no time the sign can replace the basic recommended methodological procedure.

## Conclusions

The medial meniscus flounce sign has been shown to have high diagnostic value. Use of this sign in routine knee arthroscopy can be helpful, particularly during screening procedure and in exploring tears which are usually not seen easily through routine portals.

## Abbreviations

ACL: anterior cruciate ligament
FN: false negative
FP: false positive
MRI: magnetic resonance imaging
NPV: negative predictive value
PCL: posterior cruciate ligament
PPV: positive predictive value
TN: true negative
TP: true positive

## References

[1] Kim S, Bosque J, Meehan JP, Jamali A, Marder R (2011). Increase in outpatient knee arthroscopy in the United States: a comparison of national surveys of ambulatory surgery, 1996 and 2006. J Bone Joint Surg Am.

[2] Williams AM, Myers PT, Watt MC, Hewitt BJ, Owen LE, McMeniman PJ (2006). The meniscal flounce: a valuable arthroscopic sign. Knee.

[3] Wright RW, Boyer DS (2007). Significance of the arthroscopic meniscal flounce sign: a prospective study. Am J Sports Med.

[4] Watanabe M, Takeda S, Ikeuchi H (1979). Atlas of arthroscopy.

[5] Hall FM (1978). Buckled meniscus. Radiology.

[6] Park JS, Ryu KN, Yoon KH (2006). Meniscal flounce on knee MRI: correlation with meniscal locations after positional changes. AJR.

[7] Yu JS, Cosgarea AJ, Kaeding CC, Wilson D (1997). Meniscal flounce MR imaging. Radiology.

[8] Kim BH, Seol HY, Jung HS, Cha SH, Park CM, Lim HC (2000). Meniscal flounce on MR: correlation with arthroscopic or surgical findings. Yonsei Med J.

[9] Lubowitz JH, Rossi MJ, Baker BS, Guttman D (2004). Arthroscopic visualization of the posterior compartments of the knee. Arthroscopy.

